# Hemorrhage Following Removal of the Third Tonsil*Read before the Southern Section of the American Otological, Rhinological and Laryngological Association, Atlanta, March 28, 1898.

**Published:** 1898-05

**Authors:** Ross P. Cox

**Affiliations:** Rome, Ga.


### HEMORRHAGE FOLLOWING REMOVAL OF THE THIRD TONSIL.*

By ROSS P. COX, M.D.,

ROME, GA.

In the centers of medical progress removal of adenoid vegetations from the nasopharynx is now one of the commonest and most beneficent of surgical operations. In view of its importance, knowledge of this subject has come late. Specialism had first to illumine this dark cavern and explore it with educated touch. To

*Read before the Southern Section of the American Otological, Rhinological and Laryngological Association, Atlanta, March 28, 1898.

the great body of the profession to-day this region remains, so to speak, an unknown land. With blade and scissors, fire and caustic, the average doctor will attack the faucial tonsils, but shrinks before the hidden mysteries of the nasopharynx. He has read of Luschka's wayward tonsil, but his eye has not seen nor his finger felt it. He orders mild gargles and gentle sprays. If he be over bold, he makes timid passes with weak iodine behind the soft palate. This timidity about working in the nasopharynx is not confined to ordinary practitioners. The author of a recent and most excellent "Dictionary of Treatment" and professor in a great university, gravely offers the following advice about so trivial an operation as plugging the posterior nares: "This," says he, "is one of the simplest and least painful operations, in the eye of the surgeon, until he has tried it. Having once tried it he will hesitate to repeat or recommend it." However amusing such opinions may be, I think there are few even among specialists who would not regard free and obstinate bleeding from the pharyngeal vault more seriously than from any other region of their specialty, likely to be so affected. We can astringe, cauterize, compress or even ligate the visible faucial bleeding point; we can tightly pack the nostrils, fore and aft, but I believe some of us are not prepared to apply any one of these agencies with certainty and readiness to the upper pharynx. I know that dangerous loss of blood from any cause in this region is very rare. Perhaps for this very reason one is the more apt to be caught unprepared, both in previous experience and ready means.

In two thousand consecutive operations on pharyngeal adenoids in B. Frankel's clinic at Berlin only two required tamponing for hemorrhage. I witnessed several hundred of these operations at this clinic and those of Jansen, Politzer, Chiari and others, without one severe primary bleeding. Most of these cases were operated with rather dull Gottstein curettes, rarely assisted by some biting forceps. Chiari prefers a snare, operated through the nostrils. Yet almost every recent text-book mentions excessive or fatal hemorrhage from this cause, and we hear of other cases rumored around. It is probable that no man who is much occupied with this work can escape an occasional more or less awkward experience, fraught with sleeplessness, grey hair and blood.

In my limited experience of nine such operations, my seventh gave me the greatest trouble and anxiety, though finally making a good recovery in all respects. The patient was white, female, aged thirteen years. The hemorrhage was secondary, like that of the fatal case reported by Newcomb. It occurred four days and two hours subsequent to operation and under exceedingly predisposing circumstances. The growth was large. There was middle ear catarrh at least on one side. She was a mouth-breather and had been so as far back as she could remember. The hypertrophy could be seen through the nostrils. There was chronic naso-pharyngeal catarrh, but no obstruction in the nose itself. A younger brother is similarly affected. The faucial tonsils were moderately enlarged. The constantly opened mouth detracted much from an otherwise attractive face. Cosmetic considerations, I think, weighed much in gaining consent to operation. There was no acute inflammation.

I operated without anesthesia, using an aseptic Gottstein curette and also a biting forceps. A considerable mass was removed. The primary flow of blood was only about the average. Proper directions as to antiseptics, food and exercise were given. Recovery progressed with perfect satisfaction for four days. The patient felt as well or better than usual. She forgot her precautions. With body bent forward and head low, she turned a heavy and difficult ice-cream freezer. Suddenly blood began to ooze from the nostrils and appeared in the mouth. Moderate nose-bleed was not unusual with her. Light bleeding recurred at intervals during the afternoon. I was not notified. One-half hour after retiring, eight hours after first bleeding, and four and a half days after operation an alarmingly free and persistent hemorrhage took place.

Two physicians who preceded me to the case had plugged both nostrils with cotton. After an hour's delay I arrived. Parents stated that she had lost over half a tin wash-basin of blood. Bleeding continued, but not so freely as at first. I removed the cotton and hastily directed a stream of ice-cold solution of alum and tannin through an Eustachian catheter against the pharyngeal vault. This helped, but bleeding persisted. Using a retractor, I then tried packing nasopharynx with a strip of iodoform gauze. Soon a fold of this fell down, gagged the patient, and had to be removed. Before I could proceed to pack again the bleeding lessened so much that it finally ceased under the use of cold and astringents. The patient remained with head and shoulders well elevated. Morphine was administered by the needle for shock and to secure rest.

Thirty-six hours later there was a second hemorrhage, but something less than one-half the first. It ceased before I could interfere, under the use of cold and astringents by a colleague.

Two days later, the patient still remaining in bed with elevated head and shoulders, there occurred a third and very severe loss of blood. The bleeding was progressing when I arrived. Cold and astringents did not control it. The patient was greatly reduced. I was seriously alarmed for her life. But meantime, I had devised a tampon for the nasopharynx, which worked so easily, quickly and effectively, that it is my chief excuse for mentioning this case at all. Its advantages are that it can be hastily prepared, can be used by the comparatively inexperienced and that it is effective.

It consists, as you will see, of three cords and a strip of gauze, suited to the space to be packed. The first end is introduced by one cord, as ordinarily, and is so anchored at the post-nares. At the other end of the gauze, two threads are attached. One serves merely as a tactor, as ordinarily, for removing the gauze. The other thread, after being secured at the lower end of the strip, doubly transfixes the gauze at proper intervals and finally emerges at the first end to hang free along with the first mentioned thread. The working is simplicity itself. Having applied vaseline freely to your prepared iodoform gauze and having anchored the first end at the posterior nares, precisely as in post-nasal tamponing, you easily feed up the rest of the gauze, partly by drawing on the guiding puckering string and partly with finger or instrument behind the soft palate; finally ramming it home tightly with the finger and a smart pull on the cord that fixes each separate fold and the last end of the tampon in the nasopharynx. The two fixation threads are then tied over a plug at the anterior nares.

The vaseline, or other lubricant, is to be recommended for the following reasons: It facilitates the introduction of the packing; it renders the plug more impervious to blood; by giving a smoother surface, there is less pressure reaction, but chiefly because it prevents such cohesion between the raw surface and tampon that bleeding would be renewed when the gauze is withdrawn.

I think the ideal device for the hemorrhage in question is a suitably shaped rubber bag, to be anchored and inflated by a connected tube, passing out at the anterior nares.

From six to twenty-four hours should be long enough for such a packing to remain in place.

In the presence of any hemorrhage above the clavicle, one must not overlook a possible constriction by clothing, which obstructs the return circulation. I know of one obstinate bleeding that ceased immediately on loosening a tight collar, and that, too, after all applied means had failed.

